# More Than Meets the Eye: The Impact of Materialism on Information Selection During Luxury Choices

**DOI:** 10.3389/fnbeh.2018.00172

**Published:** 2018-08-24

**Authors:** Catherine Audrin, Tobias Brosch, David Sander, Julien Chanal

**Affiliations:** ^1^Swiss Center for Affective Sciences, Geneva, Switzerland; ^2^Department of Psychology, University of Geneva, Geneva Switzerland; ^3^Research Support Centre, University for Teacher Education, Lausanne, Switzerland

**Keywords:** eye-tracker, information processing, materialism, luxury, choice

## Abstract

Visual attention is an important condition for consumer decision-making. However, not much is known on individuals' determinants of this visual attention. Using eye tracking, this study investigated how psychological values (i.e., materialism) modulate visual attention to specific sources of information (i.e., product, brand and additional information) in the context of luxury consumption. Participants were asked to perform a forced-choice experiment, where products were randomly assigned with luxury and non-luxury brands (Experiment 1) and product information (Experiment 2). Experiment 1 revealed that materialism was related to relatively higher attention to luxury as opposed to non-luxury and higher choice proportion of products displayed with a luxury brand. Experiment 2 showed that when providing additional product information (e.g., regarding the material) in addition to the brand, all participants chose luxury products more often. Interestingly, choices seemed to be driven by enhanced attention to brand for participants with high levels of materialism when choosing luxury products. In contrast, choices were driven by text for participants with low levels of materialism for non-luxury products. This suggests that individuals with high levels of materialism may prefer luxury products for different reasons than individuals with low levels of materialism: while the first focus on the symbolic dimension conveyed by the brand (Experiment 1), the latter pay attention to the actual product characteristics (Experiment 2). Taken together, our results suggest that materialism as a psychological value has an impact on visual attention and information selection during decision-making in the context of luxury consumption.

## Introduction

When you scroll down on your computer on a retailer website, what information do you attend to when trying to make your decision? Visual attention is defined as the degree to which people visually focus on a stimulus within their range of exposure (Solomon et al., 2010), and is an important precondition for product choice. Attentional mechanisms allow people to select a subset of information, while suppressing the non-selected information for further processing (Wedel and Pieters, 2008). This selection of information is a crucial step in purchase decisions (Milosavljevic and Cerf, 2008), suggesting that it may be helpful to measure visual attention and information acquisition using techniques such as eye-tracker to better understand the process leading to these kinds of decisions.

Several studies have been performed in the context of purchase decisions to investigate the role of visual attention processes. As Orquin and Mueller Loose (2013) illustrates in his review, visual attention is strongly related to eye movements. Recording eye-movement data allows to study the process of information acquisition, which is performed by eye fixations and saccades (Shi et al., 2012). Eye-tracking data thus does not only record the time spent looking at a product, but also the position and duration of each eye fixation (Chandon et al., 2009). Interestingly, previous research revealed a discrepancy between self-reported and eye-tracking measures (Graham and Jeffery, 2011), suggesting that people are usually not aware of their eye fixations (Chandon et al., 2009). Studies using eye-tracking measures have pointed out that during choice, a pre-decisional gaze bias occurs toward the preferred option (Chae and Lee, 2013). This bias, referred to as the gaze cascade (Shimojo et al., 2003), consists in a shift of attention toward the preferred choice alternative (Krajbich and Rangel, 2011; Willemsen et al., 2011). The preferred option is thus observed during a greater amount of time (Glaholt and Reingold, 2011; Glöckner and Herbold, 2011). The attentional Diffusion-Drift Model (aDDM, Krajbich and Rangel, 2011) suggests that gaze fixation is the mechanisms by which decision makers retrieve information about each option. According to this model, spending time looking at an option means that we accumulate evidence in favor of the fixated alternative (Krajbich and Rangel, 2011).

Information selection is achieved through two processes: bottom-up and top-down processes (Wedel and Pieters, 2008). Bottom-up processes correspond to a rapid and automatic way to capture attention (Milosavljevic et al., 2012). They refer to factors such as visual saliency (Glaholt and Reingold, 2011; Atalay et al., 2012; Milosavljevic et al., 2012; Janiszewski et al., 2013). In contrast, top-down processes refer to a voluntary attentional capture which requires personal and active search (Wedel and Pieters, 2008). This voluntary focus may be driven by the task, by people's previous knowledge, social identity (Xiao and Van Bavel, 2012), interests or goals (Milosavljevic and Cerf, 2008; Glöckner and Herbold, 2011). As an example of the impact of people's previous knowledge, brand usage (or familiarity with a brand) has been shown to diminish search costs for the consumer, leading them to greater effectiveness in terms of decision making (Chandon et al., 2009). Further evidence suggest that top-down processes drive attention toward the pieces of information that are relevant or critical for the ongoing decision (Ares et al., 2013; Orquin and Mueller Loose, 2013). For instance, consumers who are evaluating which product they intend to buy spend more time on text information (Rayner et al., 2001; Cisek et al., 2014), whereas consumers assessing product advertisements spend more time on pictorial information (Rayner et al., 2008). Xiao et al. (2016) suggest that social identity may tune visual attention. In their model, visual inputs are embedded with social values. As a consequence, people's social identity strongly influences their perception. Interestingly, these top-down influences may act on early attentional stages of visual perception (Brosch and Van Bavel, 2012; Xiao et al., 2016). More generally, people's motives, experiences and concerns may have an impact on their visual attention to a stimulus (Pool et al., 2016). As an example, research has revealed that participants' pro-environmental orientation lead them to have a greater propensity to attend to climate change images (Sollberger et al., 2017). Another study suggested that attention paid to nutrition labels was influenced by participants' goals (Graham and Jeffery, 2011): focusing on health goals enhanced participants' attention and intensity of processing (Visschers et al., 2010), leading to an increase of choices of healthy products (van Herpen and Trijp, 2011). Thus, literature suggest that visual attention may be influenced by top-down processes such as personal characteristics, their goals and social identity (see e.g., Pool et al., 2016).

In this study, we were interested in how materialism as a psychological value may impact visual attention to specific sources of information in the context of luxury consumption. Materialism is defined as an extensive concern for material objects and worldly possessions (Belk, 1985), and leads people to have high commitment toward the acquisition and consumption of possessions (Rindfleisch et al., 1997; Dittmar, 2005). As a value, materialism has an impact on people's goals, and is considered to be a tendency to favor extrinsic aspirations (i.e., wealth, popularity, attractiveness, and conformity) over intrinsic aspirations (i.e., self-acceptance, affiliation, community feeling, safety, spirituality, hedonism, and health). Materialistic individuals, i.e., people with high levels of materialism, are more likely to look for prestigious products reflecting a high social status (Fournier and Richins, 1991; Wang and Wallendorf, 2006) than non-materialists. More specifically, materialism is a critical dimension of luxury consumers' values (e.g., Fournier and Richins, 1991) as it enhances interest for luxury brands (Gil et al., 2012), preference for luxury goods (Wong and Ahuvia, 1998; Prendergast and Wong, 2003) and brand label consideration (Audrin et al., 2017a,b).

Brand labels are extrinsic cues (Bredahl, 2004). Extrinsic cues are any piece of information about the product that is not directly part of the product itself (Zeithaml, 1988), such as its price or the label displayed on it. Intrinsic cues refer to the physical composition of the product. Taking the example of a handbag, intrinsic cues would include the color, the texture or the material of the product (Zeithaml, 1988). Literature has shown the importance of extrinsic cues in consumer decision-making. For example, consumers' expectancies about a brand impact experienced pleasantness when consuming the product (e.g., Allison and Uhl, 1964; McClure et al., 2004). When participants were drinking Coke and Pepsi without knowing what they are drinking, experienced pleasantness was similar for both drinks. However, when drinks were labeled with a brand (Coke or Pepsi), participants reported increased preferences toward Coke (McClure et al., 2004). Thus, consumers heavily rely on extrinsic cues to build their preferences (Kuusela et al., 1998; Kardes et al., 2001; Veale and Quester, 2009; Baer et al., 2017).

Here, we report two eye-tracker studies designed to assess how materialism impacts visual attention to extrinsic (i.e., pictorial) information (Experiment 1) as well as both extrinsic (i.e., pictorial) and intrinsic (i.e., text) information (Experiment 2) that was provided for a set of products in a forced-choice task. In the first experiment, we randomly presented products with either luxurious or non-luxurious brand labels (i.e., pictorial information) to participants with high and low levels of materialism. Regarding Experiment 1, our hypotheses were that (1) all participants would choose more often products at which they look longer, (2) people scoring higher on materialism would look longer at products presented with a luxurious brand label and (3) people scoring higher on materialism would choose more often products presented with a luxurious brand. In Experiment 2 participants with high and low level of materialism were randomly presented with products in either a luxurious or a non-luxurious condition. The conditions were determined by both pictorial (i.e., brand label) and text (i.e., country of origin and material) information. Regarding Experiment 2, we hypothesized that (1) all participants would more often choose products at which they looked longer. Based on previous evidence suggesting that when providing with information about brand and quality, both participants with high and low level of materialism evaluate more positively luxury (Audrin et al., 2017a), we hypothesize that (2) all participants would show higher visual attention and choose the luxurious condition more often than the non-luxurious condition. Finally, as previous evidence reveals that materialism is related to high importance to brand labels and that people scoring low on materialism look for more information, we hypothesize that (3) pictorial information would be more determinant in the choices of participants scoring high on materialism, whereas textual information would be more determinant in the choices of participants scoring low on materialism.

## Pre-test study

We first attempted to identify luxurious and non-luxurious brands adapted to our sample. One hundred and sixty-five female students in the first year of a psychology degree at the University of Geneva were asked to name as many luxury brands as they could. Then, they were presented with 106 brands of ready-to-wear products and asked whether they knew them. These brands were selected from advertisements seen in the newspapers or on television. In addition, we selected brands mentioned in the GenY Prestige Brand Ranking (L2 Think Tank and Stren, 2010), which ranks the top luxurious brands for women. For each brand, a label was presented and participants were asked to click on “1” if they knew the brand and “0” if they did not. Based on these tests, we computed scores for each brand to select the most well-known luxurious and non-luxurious brands. Chanel®, Gucci®, Dior®, and Louis Vuitton® appeared to be the most well-known luxurious brands and H&M®, Zebra®, GAP®, and Forever21®, the most well-known non-luxurious brands. Knowledge of the brands was moreover tested on participants of the eye-tracker experiments: on average, participants knew 93.67% of the brands (*SD* = 8.25).

## Experiment 1

### Methods

#### Participants and procedures

**Figure 1:**
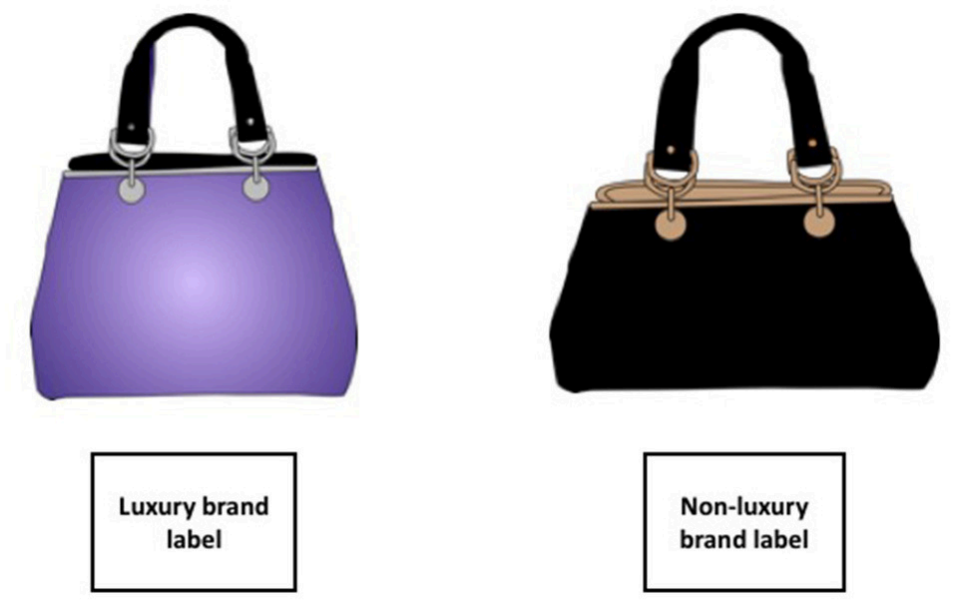
Schematic description of the task in Experiment 1 and 2.

To test the effect of luxurious vs. non-luxurious brands and its moderation by materialism on choice, 70 female psychology students at the University of Geneva were recruited for Experiment 1 (mean age = 22 ± 3 years). Our stimuli were products designed to be worn by women, we consequently only invited females to participate. Participants were first asked to complete an online full version of the Aspiration Index (Grouzet et al., 2005) in order to measure their materialism. After several weeks, all participants who completed the Aspiration Index were invited to the lab to participate to the eyetracker experiment in exchange for course credits. When coming to the laboratory, participants were invited to sit in front of a computer in a cubicle. We presented a set of 46 images of ready-to-wear products from the following categories to the participants: belts, handbags, purses and scarves. The selected products came from brands most-likely unknown to our sample (i.e., brands unknown as assessed in the pre-test study above), which ensured that the products or their brand could not be recognized by our participants. Each product was presented with 1 out of 8 brands. Half of the products were randomly presented with one out of four luxurious brand, while the other half was presented with one out of four non-luxurious brands. The luxurious and non-luxurious brands were randomized between participants so that each product was seen with each of the brands across the whole sample (see Figure 1). The study was performed according to the rules and regulations of the University of Geneva and the declaration of Helsinki. All participants gave their written informed consent to take part in the study, which was officially approved by the ethics committee of the University of Geneva.

### Data acquisition

#### Materialism

To assess participants' level of materialism, we used the full version of the Aspiration Index (Grouzet et al., 2005), which refers to Kasser (1999)'s conceptualization of materialism as a balance between extrinsic and intrinsic aspirations. In the Aspiration Index (Grouzet et al., 2005), people assessed importance of 57 aspirations on a scale ranging from 1 (“not important at all”) to 9 (“extremely important”). These goals refer to 11 aspirations, which can be separated into two main dimensions, i.e., intrinsic and extrinsic dimensions. Extrinsic dimensions refer to the importance given to one's own image (e.g., “I hope for the future that my image will be one other's find appealing”), popularity (e.g., “I will be admired by many people”), financial success (e.g., “I will have expensive possessions”), and conformism (e.g., “I will live up to the expectations of my society”). Intrinsic dimensions are the importance given to his own health (e.g., “I will feel energetic and full of life”), affiliation (e.g., “There will always be someone around to take care of me”), spirituality (e.g., “I will find personal answers to universal spiritual questions such as: Is there a supreme spiritual being? Is there life after death? What is the meaning of life?”), community (e.g., “I will assist people who need it, asking nothing in return”), hedonism (e.g., “I will have a lot of excitement in my life”), safety (e.g., “I will have few threats to my personal safety”) and self-acceptance (e.g., “I will feel free”). Materialism scores were computed as the relative importance of extrinsic (mean of the extrinsic dimensions' scores) vs. intrinsic aspirations (mean of the intrinsic dimensions' scores) (Kasser, 1999; Grouzet et al., 2005). The more people considered extrinsic aspirations important compared to intrinsic aspirations, the more they were materialists. Our sample had an average of −1.589 score on materialism (*SD* = 1.272).

#### Eye-tracking

Participants seated 60 cm away from a 43 cm-wide screen and were asked to keep their head in the same position during the experiment. Movement of the eyes were recorded at a 60-Hz frequency using infrared cameras of the Tobii eye-tracker located at the bottom of the screen. The eye-tracker was calibrated before each session. This phase consisted in the presentation of moving dots that participants were asked to follow without anticipating the dots' movement.

After this calibration, participants were presented with two ready-to-wear products side by side. For each trial, one product was presented with a luxurious brand and the other with a non-luxurious brand. The side of the luxurious/non-luxurious condition was randomized. Participants were asked to select the product they would prefer to get if they could obtain it. There was no time constraint. Each participant had to make 46 choices, where each of the 46 products was presented twice (each time with the same brand label, every time against another product). After each choice, a fixation cross was displayed in the center of the screen to wait for the next pair of products to be presented. The order of presentation was randomized between participants.

### Data analyses

Data analyses were performed with R (R Development Core Team, 2008), lmerTest (Kuznetsova et al., 2017), and lme4 packages (Bates et al., 2015). (Generalized) Linear Mixed Model ((G)LMM) analyses were performed on eyetracker and behavioral data. We used participants and stimuli as random effects (i.e., random intercepts) to control for variance due to participants and items, respectively (Judd et al., 2012). For the fixed effects, we assigned the coding −1/+1 as advised by Judd et al. (2012), which allowed us to interpret these effects as main effects. We visually inspected residual plots to detect deviations from homoscedasticity or normality. Descriptive analyses (mean and s.e.) are reported bellow. Concerning the eye-tracker data, analyses were performed on the total fixation time. Due to its non-normal distribution, fixation time was log-transformed, but for the ease of interpretation, means were back-transformed for Table 1. Our model contained Condition (luxurious vs. non-luxurious condition), Materialism and Information (product vs. brand) as fixed effects. Concerning the behavioral data, we performed analyses on participants' choices (Table 2. For each choice, we predicted the probability that the product on the left would be chosen. Selecting the product on the left or on the right as a dependent variable is equivalent, as the position (right or left) of the luxurious and non-luxurious brands was randomized. To analyse this dichotomous variable, we performed a multilevel logit model. The fixed effects were Condition (luxurious vs. non-luxurious condition), Materialism (materialist vs. non-materialist) and the Time (time spent before making the choice in milliseconds). Descriptive statistics for the choice variable are reported in Table 2.

**Table 1:** Descriptive statistics for the Total Fixation Time variable (mean and s.e.).

**Materialism**	**Condition**	**Information**	**Time (ms)**	**s.e**.
−1 s.d.	Luxury	Image	958.337	84.227
−1 s.d.	Luxury	Brand	176.542	11.632
−1 s.d.	Non-luxury	Image	957.088	80.370
−1 s.d.	Non-luxury	Brand	182.390	11.626
mean	Luxury	Image	944.758	56.846
mean	Luxury	Brand	179.456	7.850
mean	Non-luxury	Image	929.435	54.242
mean	Non-luxury	Brand	184.375	7.847
+ 1 s.d.	Luxury	Image	932.756	79.051
+ 1 s.d.	Luxury	Brand	182.032	10.917
+ 1 s.d.	Non-luxury	Image	904.994	75.431
+ 1 s.d.	Non-luxury	Brand	186.128	10.912

**Table 2:** Descriptive analyses for the Choice variable (mean and s.e.).

**Materialism**	**Condition**	**Proportion of choice**	**s.e**.
−1 s.d.	Luxury	50.8%	0.023
−1 s.d.	Non-luxury	49.12%	0.023
mean	Luxury	51.58%	0.016
mean	Non-luxury	48.41%	0.017
+1 s.d.	Luxury	52.29%	0.023
+1 s.d.	Non-luxury	47.70%	0.023

### Results

#### Total fixation time

Results for the total fixation time showed a main effect of Information [*b* = 0.787, *IC*_95%_ = [0.763; 0.812], *t*_(8, 125)_ = 62.992, *p* < 0.001; see Table 3], revealing that participants spent more time looking at the product picture than at the brand. The Condition × Materialism interaction effect was significant [*b* = −0.013, *IC*_95__%_ = [−0.026; −0.001]; *t*_(8, 089)_ = −2.047, *p* = 0.041], supporting our hypothesis that materialism had an impact on the time spent on luxury vs. non-luxurious condition. Interestingly, results revealed that the more participants are materialistic, the less they look at non-luxury. There was no further significant effect.

**Table 3:** Results for the Total Fixation Time variable, ^***^p < 0.001; ^*^p < 0.05.

**Fixed Effects**	**b**	**SE**	***p*-value**
Intercept	0.579	0.002	0.001^***^
Condition	0.017	0.012	0.137
Information	0.787	0.013	0.001^***^
Materialism	0.018	0.038	0.641
Condition × Information	0.005	0.012	0.629
Condition × Materialism	−0.013	0.006	0.041^*^
Information × Materialism	−0.001	0.003	0.261
Condition × Information × Materialism	−0.001	0.008	0.801
**Random effects**	σ^2^	**SE**	
**Participants**
Intercept	0.153	0.391	
**Stimuli**
Intercept	0.003	0.609	

#### Choice

Results showed a main effect of Condition (*b* = 0.232, *IC*_95__%_ = [0.112; 0.354], *z* = 3.771, *p* < 0.001; Table 4), where products associated with luxurious information were more often chosen than products associated with non-luxurious information. The marginal effect of Time (*b* = 0.051, *IC*_95__%_ = [-0.000; 0.103], *z* = 1.941, *p* = 0.052) supported the hypothesis that the more people looked at the product, the more they chose it. Finally, the Condition × Materialism interaction was significant (*b* = 0.086, *IC*_95__%_ = [0.023; 0.148], *z* = 2.708, *p* = 0.006), supporting the hypothesis that the impact of materialism depended on the product: the more participants were materialistic, the more they would choose luxury products over non-luxury products (*b* = 0.12, *z* = 2.562, *p* < 0.001).

**Table 4:** Results for the Choice variable, ^***^p < 0.001; ^*^p < 0.05.

**Fixed Effects**	**b**	**SE**	***p*-value**
Intercept	−0.318	0.128	0.013^*^
Materialism	−0.037	0.036	0.304
Condition	0.232	0.062	0.001^***^
Time	0.051	0.026	0.052.
Condition × Materialism	0.086	0.032	0.006^***^
**Random Effects**	σ^2^	**SE**	
**Participants**			
Intercept	0.041	0.202	
**Stimuli**			
Intercept	0.398	0.631	

### Discussion

The results presented above revealed that the longer participants looked at a product, the higher the probability that this product was chosen. While the result is marginally significant in our study, it is congruent with previous evidence (Glaholt and Reingold, 2011; Atalay et al., 2012; Milosavljevic et al., 2012) suggesting that higher attention to a product is driven by enhanced interest (Fink et al., 1996), thus leading to enhanced preference. Our results reveal for the first time that this is also true in the context in luxury consumption.

Our results further suggest that despite their longer attention to the product itself compared to the extrinsic cues, participants still integrated extrinsic cues (i.e., brand label) into their choices. The more participants were materialistic, the less they looked at products displayed with a non-luxurious brand. These results revealed that the mere association of a brand with a product can lead participants to look at it differently, and to choose it differently, depending on their materialism. This is in line with previous evidence revealing the importance of external cues in consumer choices (e.g., McClure et al., 2004), but we revealed here that this can also be observed in the context of luxury consumption.

Finally, our results reveal that psychological values such as materialism modulate the impact of extrinsic cues (e.g., Sörqvist et al., 2015). While the effect was not strong in our results regarding the pattern of visual attention, results on the choice variable revealed that the brand had a different impact on participants' choice, depending on their level of materialism. These results show further evidence that the brand was a strong vector of the luxury dimension of a product for materialistic people (Audrin et al., 2017a,b). More generally, this result echoes previous results revealing the strong link between materialism and luxury (Gil et al., 2012).

In the next experiment, we studied how providing textual information about the product in addition to the brand label modulates participants' visual attention and subsequent choices. We hypothesize that, in contrast to Experiment 1, where only brand information was provided, providing supplementary information about the product quality will lead both participants with high and low levels of materialism to prefer luxury.

## Experiment 2

Products are embedded with multiple cues (Miyazaki et al., 2005) such as quality information and brands information. When facing multiple congruent cues, people usually take them into account in an additive way (Anderson, 1981; Miyazaki et al., 2005). However, research suggest that individual characteristics may impact the way multiple cues are integrated in the process of decision-making. For instance, Ahluwalia et al. (2000) revealed that depending on their brand commitment, consumers gave different diagnostic weights for positive and negative information.

In this experiment, we wanted to assess how materialism as a value impacts information integration during choice. To this end, we provided text information in addition to the brand such as the country of origin and the material of the products. Our hypothesis was that when provided with supplementary intrinsic information, both participants with high and low materialism will prefer luxury. We suggest however that this may be driven by different reasons: materialistic participants can be expected to predominantly consider the symbolic aspect embedded in luxury, as previous research has shown that people high in materialism focused on the brand in the context of luxury consumption (Gil et al., 2012; Audrin et al., 2017a). Thus, we hypothesize that, as in Experiment 1, people with high level of materialism would primarily focus on the brand. In contrast, we hypothesize that consumers with low levels of materialism would primarily look for more objective information about the product as provided by the textual information, as literature suggests that they like luxury preferably for the quality it guarantees (Audrin et al., 2017a). Thus, we suggest that people low in materialism will mostly look at intrinsic textual information about the product. Taken together, we hypothesized that (1) all participants would show higher visual attention and choose luxurious condition more often than non-luxurious condition (Model 1), (2) all participants would choose more often products at which they looked longer (Model 2) and finally (3) pictorial information would be more determinant in the choices of people with high level of materialism, whereas textual information would be more determinant in people's with low level of materialism choices (Model 2).

### Methods

#### Participants and procedure

Among 195 female students in psychology at the University of Geneva, 60 were recruited based on their materialism scores on the Aspiration Index (Grouzet et al., 2005). Four of them were removed from the final sample because the eye-tracker was not able to detect their eyes, resulting in a sample of 56 participants (*mean age*: 22 ± 4 years). Our final sample consisted of 24 people with high levels of materialism (*mean* = 0.29 ± 0.42) and 32 people with low levels of materialism individuals (*mean* = −2.55 ± 0.52).

When coming to the laboratory, participants were invited to sit in front of a computer in a cubicle. The same set of 46 images of ready-to-wear products were presented as in Experiment 1. Half of the products were randomly assigned to in the luxury condition, while the other half was presented in the non-luxury condition. The luxury and non-luxury conditions were randomized between participants so that each product was seen with each of the brands across the whole sample. The eye-tracker was calibrated before each session. The task was similar to the one described in Experiment 1. In order to test the interaction between Materialism, Condition and Information, we manipulated the text information provided with each product. Each product was presented with a brand, as well as with information about the product's price, country of origin, and material. This information was gathered from the websites of the brands in question, and adjusted according to the presented product. Half of the products were presented in the luxurious condition (i.e., country of origin, price, material), while the other half was presented in the non-luxurious condition. The number of words was the same in the text associated with luxurious and non-luxurious brands (3 words for the country where the product was made and 2 words for the composition; see Figure 3 for a prototypical example). The study was performed according to the rules and regulations of the University of Geneva and the declaration of Helsinki. All participants gave their written informed consent to take part in the study.

### Data acquisition

#### Materialism

One hundred and ninety-five participants were presented with the Aspiration Index, and we computed their materialism scores as the relative importance of extrinsic aspirations vs. the importance of intrinsic aspirations. Based on these materialism scores for all participants, we selected the upper (high level of materialism) and lower (low level of materialism) quartile of the initial sample to participate to the experiment.

#### Eye-tracking data

When coming to the laboratory, participants were seated 60 cm away from a 43 cm-wide screen and were asked to keep their head in the same position during the experiment. As for Experiment 1, movement of the eyes were recorded at a 60-Hz frequency using infrared cameras of the Tobii eye-tracker, located at the bottom of the screen.

### Data analyses

To answer our first hypothesis (i.e., that all participants will show higher visual attention and choose luxurious condition more often than non-luxurious condition), we performed a linear mixed model on the total fixation time (Model 1). We entered participants and stimuli as random effects. As fixed effects, we introduced Information (i.e., picture vs. text information), Condition (luxurious vs. non-luxurious condition), and Materialism (high vs. low materialism) and their interaction. Descriptive statistics are reported in Table 5.

**Table 5:** Descriptive statistics (mean and s.e.) for the Total fixation time variable.

**Materialism**	**Information**	**Condition**	**Time (ms)**	**s.e**.
High	Text	Luxury	632.693	50.735
High	Picture	Luxury	1136.966	52.348
High	Text	Non-luxury	524.014	53.117
High	Picture	Non-luxury	1095.82	46.878
Low	Text	Luxury	691.535	41.558
Low	Picture	Luxury	1189.684	38.569
Low	Text	Non-luxury	623.427	47.714
Low	Picture	Non-luxury	1190.373	47.756

In order to assess our second (i.e., all participants will choose more often products at which they looked longer) and third hypotheses (pictorial information will be more determinant in participants high on materialism, whereas textual information will be more determinant in the choices of participants with low levels of materialism, respectively), a generalized linear mixed model was performed on the choices made by participants (Model 2). For each choice, we predicted the probability that the product on the left would be chosen. The fixed effects were Condition (luxurious vs. non-luxurious condition) and Materialism (high vs. low on materialism). We further introduced two variables referring to the time spent on the information variable. The first variable accounted for the relative time spent on the picture (i.e., product and brand) on the left as opposed to the total time spent on the pictorial information before making the choice (Picture Time). The second variable accounted for the relative time spent on the text information of the product on the left as opposed to the total time spent on the text information before making the choice (Text Time). Participants and stimuli were introduced as random effects (random intercepts). Descriptive statistics are reported on Table 6.

**Table 6:** Descriptive analyses (mean and standard error (s.e.)) for the Choice variable.

**Materialism**	**Condition**	**Proportion of choice**	**s.e**.
High	Luxury	55.8%	0.034
High	Non-luxury	44.1%	0.034
Low	Luxury	55.5%	0.030
Low	Non-luxury	44.4%	0.030

### Results

#### Total fixation time

**Figure 2:**
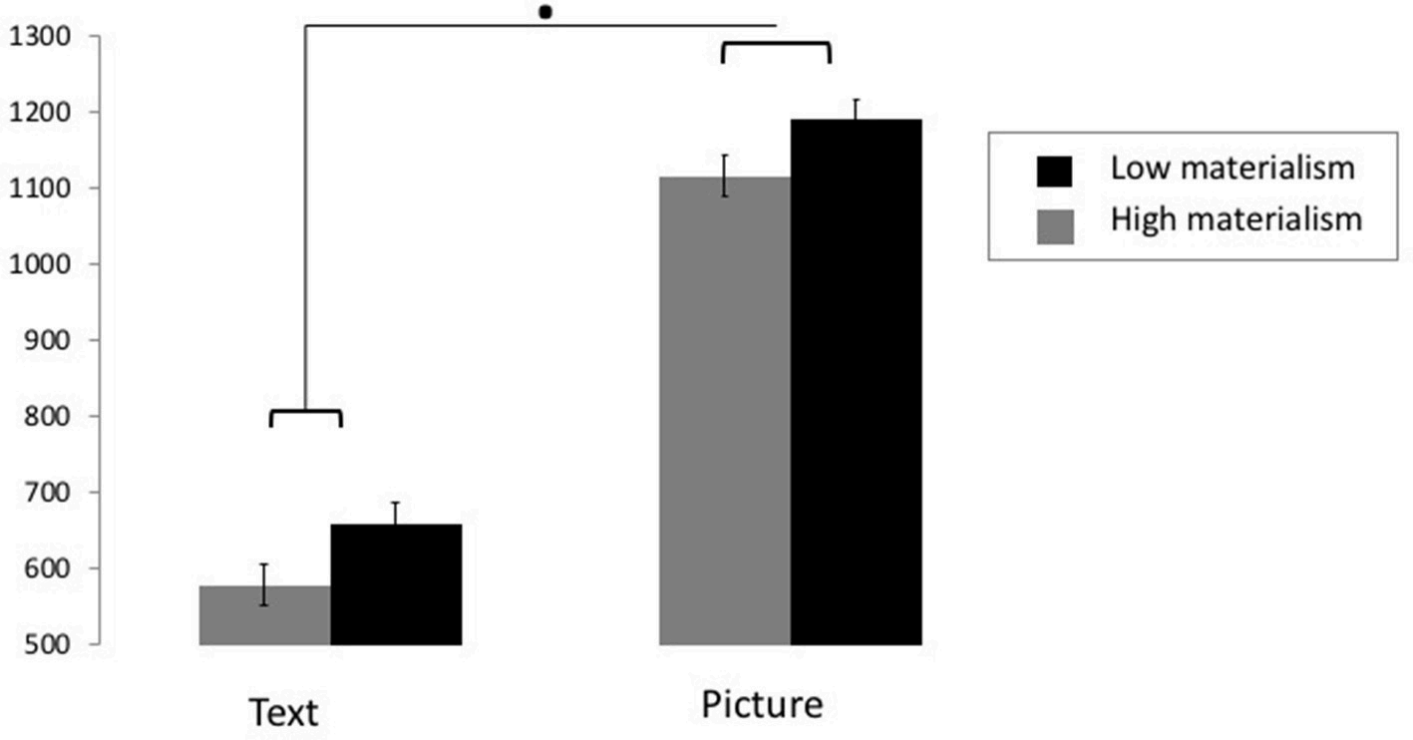
Time spent looking (mean ± SEM) as a function of the Information (text vs. picture) for participants with high (gray) and low (black) levels of materialism (black). ·*p* < 0.05.

Results of Model 1 showed a main effect of Information [*b* = −0.146, *IC*_95__%_ = [−0.155; −0.138], *t*_(7, 048)_ = −34.366, *p* < 0.001], revealing that participants spent more time looking at the pictorial elements than at the text information. Results further revealed a main effect of Condition [*b* = 0.016, *IC*_95__%_ = [0.007; 0.023], *t*_(6, 994)_ = 3.913, *p* < 0.001; Table 7], supporting our hypothesis that all participants looked more at luxurious products than at non-luxurious products. The Condition × Information interaction was significant [*b* = −9.25 ^*^ 10^−3^, *IC*_95__%_ = [−0.017; −0.001]; *t*_(6, 994)_ = 2.305, *p* = 0.023], showing that the difference between the time spent looking at luxurious and non-luxurious products was more important when participants focused on text information (*t* = −3.688, *p* < 0.001) than when they looked at the picture (*t* = −1.495, *p* > 0.05). The Information × Materialism interaction was marginally significant [*b* = −8.069 ^*^ 10 ^−3^, *IC*_95__%_ = [−0.016; 0.003], *t*_(6, 997)_ = 2.305, *p* = 0.058, see Figure 2], suggesting that the impact of materialism on time spent looking at picture vs. text was different. Specifically, this suggests that for both groups, the time spent looking at the picture was longer than the time spent looking at the text, but that this difference was slightly more important for participants with high levels of materialism. Results revealed no further significant effect.

**Table 7:** Results for the Total fixation time variable, ^***^p < 0.001; ^*^p < 0.05.

**Fixed effects**	**b**	**SE**	***p*-value**
Intercept	2.810	0.024	0.001^***^
Condition	0.016	0.004	0.001^***^
Information	−0.147	0.002	0.001^***^
Materialism	−0.001	0.023	0.795
Condition × Information	−0.009	0.004	0.023^*^
Condition × Materialism	0.005	0.004	0.253
Information × Materialism	−0.008	0.004	0.059.
Condition × Information × Materialism	0.002	0.004	0.485
**Random effects**	σ^2^	**SE**	
**Participants**
Intercept	0.03	0.17	
**Stimuli**
Intercept	0.001	0.041	

#### Choice

**Figure 3:**
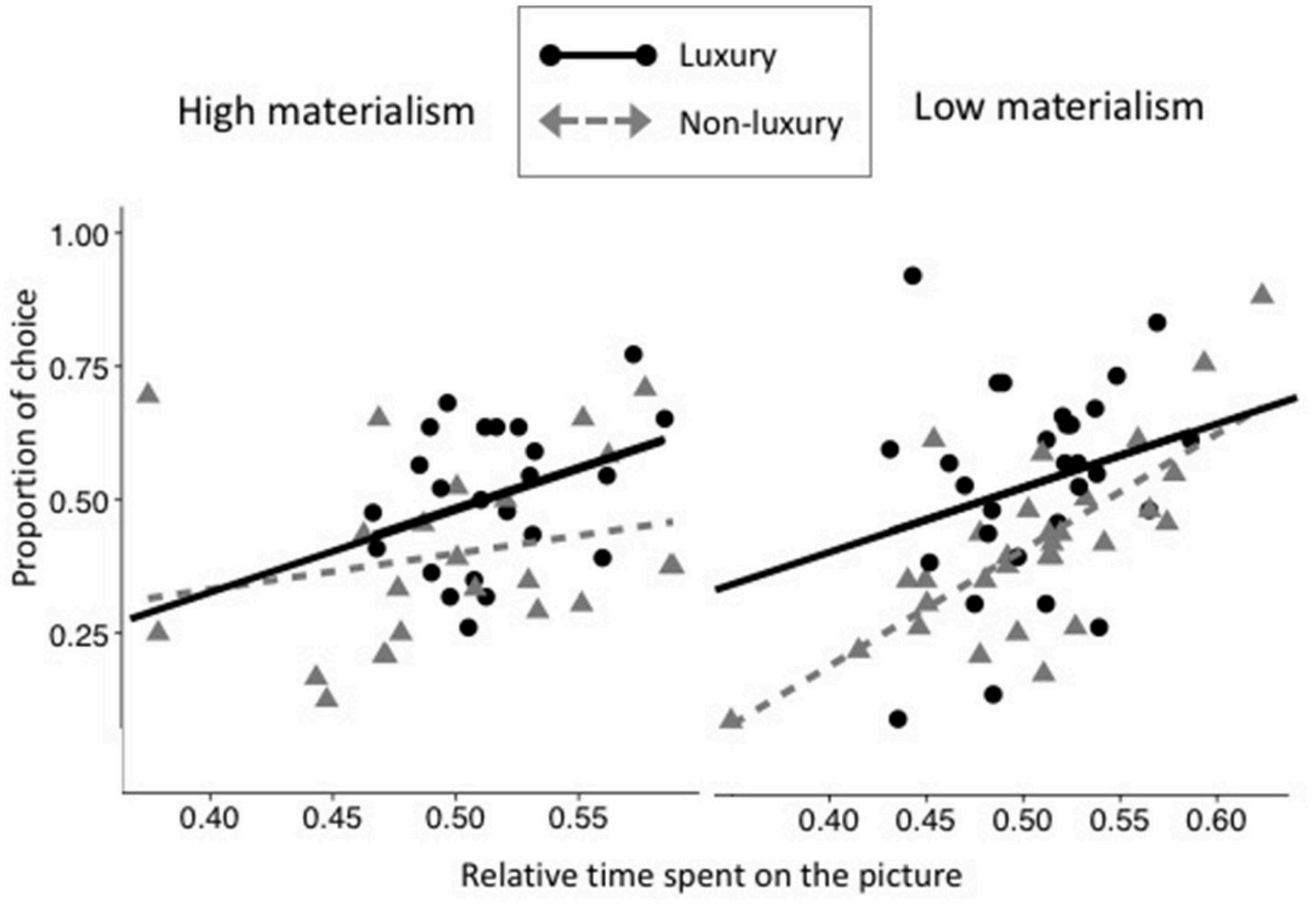
Proportion of choices of the object as a function relative time spent looking at the picture in luxury (black plain line and circles) and non-luxury condition (gray dashed lines and triangles). Left panel depicts people with high levels of materialism and right panel depicts participants with low levels of materialism.

Results from Model 2 (i.e., with Condition, Materialism and Time) showed a main effect of Condition (*b* = 0.258, *IC*_95__%_ = [0.169; 0.349], *z* = 5.640, *p* < 0.001; Table 8), where luxury products were chosen more often than non-luxurious products. The main effects of Picture Time (*b* = 3.547, *IC*_95__%_ = [2.936; 4.153], *z* = 11.429, *p* < 0.001) and of Text Time (*b* = 1.079, *IC*_95__%_ = [0.780; 1.388], *z* = 6.957, *p* < 0.001; see Table 8) revealed that, as hypothesized, the more participants looked at the text and pictorial information, the more they chose it. The interaction between the Picture Time and Text Time was significant (*b* = −1.904, *IC*_95__%_ = [−3.656; −0.059], *z* = −2.033, *p* = 0.042), indicating that the impact of the time spent looking at the picture on the choice weakened when the time spent looking at the text increased. Moreover, the interaction effect between Condition, Materialism and Picture Time was marginally significant (*b* = 0.582, *IC*_95__%_ = [−0.024; 1, 469], *z* = 1.915, *p* = 0.055; Figure 3). This interaction revealed that when participants with low levels of materialism looked longer at non-luxurious picture, they chose them more often (*z*_low−materialism_ = 5.325, *p* < 0.001). On the other hand, participants with high levels of materialism who looked longer at the picture of luxurious condition chose them more often (*z*_highmaterialism_ = 5.794, *p* < 0.001).

**Table 8:** Results for the Choice variable (Model 2), ^***^p < 0.001; ^**^p < 0.01; ^*^p < 0.05.

**Fixed effects**	**b**	**SE**	***p*-value**
Intercept	−0.197	0.104	0.057
Materialism	−0.09	0.056	0.099.
Condition	0.258	0.046	0.001^***^
Picture time	3.547	0.31	0.001^***^
Text time	1.079	0.155	0.001^***^
Condition × Materialism	−0.047	0.047	0.317
Materialism × Picture Time	0.081	0.305	0.791
Condition × Picture Time	−0.100	0.304	0.743
Materialism × Text Time	−0.107	0.149	0.476
Condition × Text Time	−0.027	0.143	0.853
Picture Time × Text Time	−1.904	0.937	0.042^*^
Materialism × Condition × Picture Time	0.582	0.304	0.055.
Materialism × Condition × Text Time	−0.386	0.143	0.006^**^
Condition × Picture Time × Text Time	1.239	0.938	0.186
Materialism × Picture Time × Text Time	0.282	0.934	0.763
Condition × Materialism × Text Time × Picture Time	0.144	0.935	0.878
**Random effects**	σ^2^	**SE**	
**Participants**
Intercept	0.05	0.241	
**Stimuli**
Intercept	0.14	0.377	

**Figure 4:**
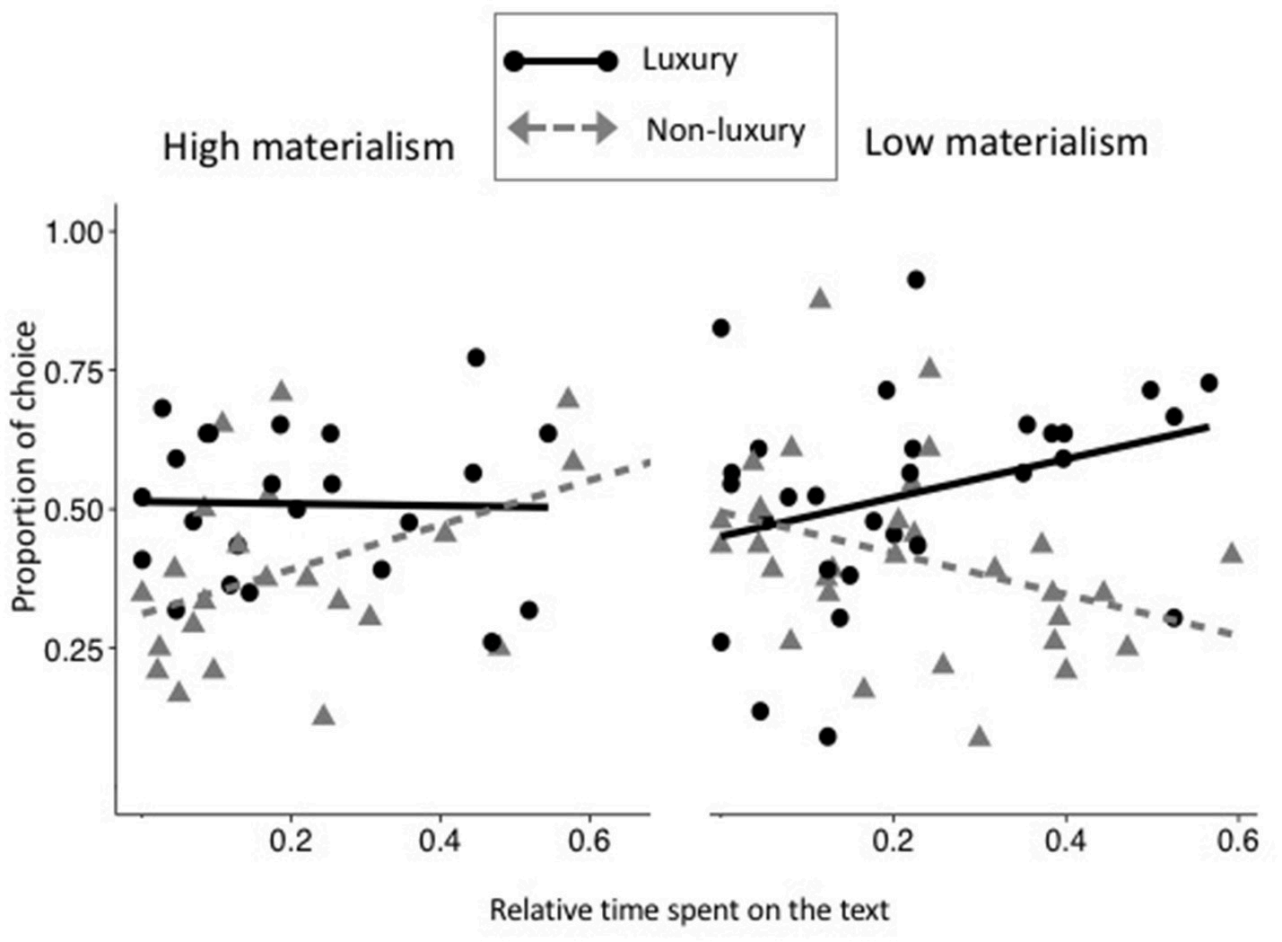
Proportion of choices of the object as a function of relative time spent looking at the text in luxury (black plain line and circles) and non-luxury condition (gray dashed lines and triangles). Left panel depicts people with high levels of materialism and right panel depicts participants with low levels of materialism.

Finally, the interaction effect between Condition, Materialism and Text Time was significant (*b* = −0.386; *IC*_95__%_ = [−0.660; −0.09], *z* = −2.702, *p* = 0.006; Figure 4). This interaction revealed that when participants who scored high on materialism looked longer at text information of non-luxurious products, they would choose these products more often (*z* = 1.882, *p* = 0.059). In contrast, when participants scoring low on materialism looked longer at the text information of luxurious products, they would choose these products more often (*z* = 5.233, *p* < 0.001).

### Discussion

Supporting our hypothesis, results from Experiment 2 showed that when providing multiple congruent sources of information about a product, all participants chose luxury products more often. Our results highlight that individual characteristics have an impact on how these multiple sources of information are taken into consideration when making a choice (Ahluwalia et al., 2000). Eye-tracking data revealed different patterns of visual attention for the groups: the more participants with high levels of materialism paid attention to the *picture* of the product (i.e., brand and picture), the more often they chose luxury products. On the contrary, the more participants with low levels of materialism looked at the *text* information, the more often they chose luxury products. These results suggest that individual values and goals lead to different patterns of visual attention and information integration during decision-making and product choice. Participants scoring high on materialism considered specifically the symbolic aspect embedded in luxury (i.e., they mostly focus on the brand): the more they looked at pictorial information, the more they chose luxury products. Participants scoring low on materialism looked for textual information about the product: the longer they looked at text information, the more they chose luxury products. This suggests that the preference for luxury may be related to specific dimension of luxury (Audrin et al., 2017a) depending on peoples' values. People with high levels of materialism favor symbolic dimensions by focusing on pictorial brand-related element (Audrin et al., 2017a,b). By contrast, participants with low levels of materialism pay relatively more attention to factual product information (Audrin et al., 2017a).

## General discussion

In this work, we investigated how materialism modulates visual attention to specific sources of information in the context of luxury consumption. Using eye-tracking, we tested how visual attention allocated to pictorial brand information (Experiments 1, 2) and textual quality information (Experiment 2) was related to product choices for participants with high and low levels of materialism.

Experiment 1 tested the importance of pictorial brand-related information. Results revealed that higher levels of materialism were related to higher visual attention to the luxurious condition (i.e., product and brand). Increased visual attention furthermore enhanced the probability of choosing luxury products. Experiment 2 tested the importance of both pictorial and textual information, revealing that when adding supplementary textual information about the products, all participants paid increased attention and chose the luxurious condition more often. Differences in materialism may have lead participants to choose luxurious products for different reasons: the longer participants with higher levels of materialism looked at pictorial information, the more often they choose luxury products. In contrast, the longer participants with low levels of materialism looked at textual information, the more they choose luxury products.

Our results provide congruent evidence for the gaze cascade effect suggested by Shimojo et al. (2003). As suggested by (Shimojo et al., 2003), eye movements and eye gaze reveal a shift of attention toward one alternative (Krajbich and Rangel, 2011), and are involved in preference formation. This preference formation may further indicate interest toward the observed alternative (Shimojo et al., 2003). Our results are congruent with this literature, as the more participants looked at a product or its feature, the more frequently they chose it. Moreover, our results reveal that materialism, as a psychological value, may have an impact on visual attention. This is congruent with previous evidence revealing the importance of top-down processes, notably the importance of people's motives, experiences and concerns, on visual attention (Pool et al., 2016).

Results from a retail context (Chandon et al., 2009) suggest that gaze may be an indicator of *attention*. On the other hand, Shimojo et al. (2003) suggest that this may be an indicator of *interest*. Finally, Fink et al. (1996) suggest that *interest* may lead to higher *attention* toward an option. Our results seem to provide congruent results with Fink et al.'s work: while previous research has suggested that materialism was related to higher *interest* toward luxury (Gil et al., 2012), our result reveal that this interest was related to higher *attention* to luxury. Future research should however specifically test the relation between materialism, interest and attention toward luxury.

As pointed out in the literature, when making a decision, individuals use attribute information as a way to estimate their probable satisfaction with the choices they are facing. However, products are often embedded with multiple attributes, which makes the integration of all pieces of information too difficult and too costly in terms of cognitive processes. People often focus on one single source of information, in order to make their decision easier (Johnson et al., 2012). This strategy allows consumers to reduce the cognitive effort, and to specifically focus on the attribute they perceive as the most important (Johnson et al., 2012). Our results suggest that this strategy was also used in our experiments, and further highlights that psychological values modulated the selection of the most important attribute.

Our findings support the suggestion that materialism, measured as a psychological value, might lead to a propensity to attend to specific aspects of luxury. As the previous literature reveals, materialistic people focus on the conspicuous aspects provided by luxury possessions (Gil et al., 2012; Audrin et al., 2017a), further suggesting that they give importance to the brand as it can be displayed overtly. Conversely, people with low levels of materialism may consume luxurious products because of their quality (Audrin et al., 2017a).

Taken together, our results provide new insights to the field of research on consumer decision-making in the context of luxury consumption. To the best of our knowledge, our study is the first to manipulate displayed brands and text information of ready-to-wear products in an eye-tracking setting. Our results provide evidence for the importance of the brand in consumers' preferences establishment. Notably, our results show how strong the impact of brand information may be to (materialistic) consumers, as the brands were randomly presented with products, but still lead to a preference for the respective products. This result, consistent with previous research (e.g., Audrin et al., 2017a,b), points out that values are important factors to be taken into account when studying consumer decision-making. While the behavioral (choice variable) results revealed strong evidence in favor of our hypotheses, evidence was weaker regarding the eye-tracking data, which gave only moderate support to our hypotheses. This kind of discrepancy between self-reported and eye-tracking measures has been previously shown in the literature (Graham and Jeffery, 2011). However, it suggests that these results should be taken cautiously, and that the experiments reported here should be replicated in order to show more compelling evidence regarding the impact of personal values on visual attention in the context of luxury consumption. The measure of fixation duration should also be taken cautiously. Indeed, Krasich et al. (2018) revealed that mind wandering (i.e., the tendency to zone out, when the attention shifts away from the on-going task toward unrelated thoughts such as grocery shopping or upcoming vacations) was related to longer fixation durations, thus suggesting that longer fixation time is not a necessarily a proof of enhanced attention or interest.

Two additional limitations of our study are related to our sample. First, our participants were exclusively students, who may not be very familiar with luxury consumption, as luxury products are high-priced and thus not affordable to students. It would be interesting to evaluate the extent to which our results may apply to actual luxury consumers. Second, we focused our research on female participants. This was related to the products chosen, which were exclusively designed for women. An interesting follow-up would be to evaluate whether the results observed in our study are also true for men. Indeed, as Stokburger-Sauer and Teichmann (2013) mentioned, female participants have more positive attitudes toward luxury brands. Thus, assessing how men are sensitive to brands vs. textual information may provide further explanations on what people look for when consuming luxury.

## Conclusion

Our results point out how psychological values (i.e., materialism) modulate visual attention to specific sources of information (i.e., pictorial brand vs. text quality information). Eye-tracking data revealed that levels of materialism modulate the importance allocated to specific sources of information in the process of luxury decision-making: when only brand information was available, materialism was related to enhanced preference for luxury. However, when supplementary information about product quality was available, all participants tended to choose luxury more often. Interestingly, while people with high levels of materialism focused on the product picture and the brand, participants with low levels of materialism focused more on the text information about product quality when choosing luxury products. To summarize, our results suggest that materialism as a psychological value may impact how visual attention is directed toward specific sources of information in the context of luxury consumption.

## Data availability statement

The raw data supporting the conclusions of this manuscript will be made available by the authors, without undue reservation, to any qualified researcher.

## Author contributions

CA, TB, DS, and JC contributed conception of the study. CA designed the study, organized the database, performed the statistical analysis, wrote the first draft of the manuscript. TB, DS, and JC contributed to manuscript revision, read and approved the submitted version.

### Conflict of interest statement

The authors declare that the research was conducted in the absence of any commercial or financial relationships that could be construed as a potential conflict of interest.
